# Salidroside Attenuates Concanavalin A-Induced Hepatitis via Modulating Cytokines Secretion and Lymphocyte Migration in Mice

**DOI:** 10.1155/2014/314081

**Published:** 2014-04-07

**Authors:** Baoji Hu, Yun Zou, Shanshan Liu, Jun Wang, Jiali Zhu, Jinbao Li, Lulong Bo, Xiaoming Deng

**Affiliations:** ^1^Department of Anesthesiology, Changhai Hospital, Second Military Medical University, 168 Changhai Road, Shanghai 200433, China; ^2^Department of Anesthesiology, Shanghai Pudong Hospital, Fudan University Pudong Medical Center, 2800 Gongwei Road, Huinan Town, Pudong, Shanghai 201399, China; ^3^Department of Anesthesiology, Chenggong Hospital, Xiamen University, 94-96 Wenyuan Road, Xiamen 361003, China

## Abstract

Salidroside, isolated from the medicinal plant *Rhodiola*, was reported to serve as an “adaptogen.” This study was designed to explore the protective effect of salidroside on concanavalin A- (Con A-) induced hepatitis in mice and investigate potential mechanisms. C57BL/6 mice were randomly divided into control group, Con A group, and salidroside group. Salidroside (50 mg/kg) was injected intravenously followed by Con A administration. The levels of ALT, AST, inflammatory cytokines and CXCL-10 were examined. The pathological damage of livers was assessed, the amounts of phosphorylated I**κ**B**α** and p65 were measured, and the numbers of CD4^+^ and CD8^+^ T lymphocytes in the blood, spleen and infiltrated in the liver were calculated. Our results showed that salidroside pretreatment reduced the levels of ALT, AST dramatically and suppressed the secretion of proinflammatory cytokines through downregulating the activity of NF-**κ**B partly. Salidroside altered the distribution of CD4^+^ and CD8^+^ T lymphocyte in the liver and spleen through regulating CXCL-10 and decreased the severity of liver injuries. In conclusion, these results confirm the efficacy of salidroside in the prevention of immune mediated hepatitis in mice.

## 1. Introduction

Hepatitis represents a major threat to human health worldwide. Autoimmune hepatitis (AIH) is a severe form of hepatitis, characterized by progressive destruction of hepatic parenchyma and cell infiltration. It was estimated that the prevalence of AIH ranges between 50 and 200 cases per million people. Without intervention, nearly 50% of patients with severe AIH die in approximately 5 years [1]. Previous studies indicated that inflammatory cytokines and T lymphocytes play an important role in the pathogenesis of AIH [2, 3]. In studying AIH, Concanavalin A- (Con A-) induced hepatitis is a well-established mouse model of immune-mediated liver injury that resembles AIH occurring in human [4]. The Con A model is a well-established model for investigating T cell dependent liver injury in mice, which closely mimics the pathogenic mechanisms and pathological changes of patients with AIH [5, 6]. Con A administration could provoke T cells activated in abundance, resulting in production and secretion of a series of inflammatory cytokines and chemokines, that is, TNF-*α*, IFN-*γ*, IL-6, and CXCL-10. These factors will exacerbate the recruitment and activation of immune cells infiltrating, leading to severe hepatic damage.

Salidroside, isolated from the medicinal plant* Rhodiola rosea*, is a phenylpropanoid glycoside. Several experimental and clinical studies of salidroside have provided evidence for its pharmacological activities, including anti-inflammation [7], antioxidation [8], antistress, antihypoxia, anticancer, and enhancing immune effects [9–11]. One recent study demonstrated that salidroside was an effective suppressor of inflammation by focusing on the prophylaxis effect of mastitis [12].

The above observations influence us to investigate whether salidroside can attenuate Con A-induced hepatitis. Therefore, the aim of our present study was to investigate the immunomodulatory effect of salidroside and explore its potential mechanisms in the murine model of Con A-induced hepatitis.

## 2. Material and Methods

### 2.1. Chemicals and Reagents

Salidroside (CAS number: 10338-51-9, purity >99%, molecular formula: C_14_H_2_O_7_, molecular weight: 300.30) was purchased from Shanghai Ronghe Medicine Science and Technology Development Co., Ltd. (Shanghai, China). Con A was purchased from Solarbio Corporation (Beijing, China). All of the other chemicals and reagents were standard commercially available biochemical quality. Deionized water was purified with a Milli-Q purification system and was used to prepare all solutions.

### 2.2. Animals

C57BL/6 male mice (aged 6–8 weeks; 20–25 g) were obtained from the Animal Experimentation Center of the Second Military Medical University. Mice were housed under specific pathogen-free condition and provided a standard laboratory chow and water freely one week before experiment. All experiments were performed in accordance with the guidelines of Institutional Animal Ethics Committee of the Second Military Medical University (Shanghai, China).

### 2.3. Experimental Design

Mice were randomly divided into three groups: control group, Con A group, and salidroside pretreatment group. Salidroside and Con A were both dissolved in normal saline. Mice were administrated with normal saline (100 *μ*L) or salidroside (50 mg/kg, 100 *μ*L) through the tail vein according to the groups, followed by a dose of Con A (20 mg/kg, 100 *μ*L) intravenously in Con A group and salidroside pretreatment group 1 h later, respectively. The protocol methods of salidroside administration were carried out following previous studies [7, 13, 14]. All dosages were determined by preliminary experiments. Blood, liver tissue, and spleen were harvested 12 h after Con A administration.

### 2.4. Liver Function and Cytokines Assay

Mice were anesthetized by sevoflurane and sacrificed to collect blood from heart. Plasma was separated after centrifugation at 300 g for 5 min. Levels of ALT and AST were measured by automatic dry biochemical analyzer (Hitachi Auto Analyzer 7170, Japan). The concentrations of TNF-*α*, IL-6, IL-10, and IFN-*γ* were detected by using enzyme-linked immunosorbent assay (ELISA) kits according to the manufacturer's instructions (R&D system, USA).

### 2.5. Histopathology Assay

Liver tissues were harvested 12 h after Con A administration intravenously. Liver samples were fixed in 4% buffered paraformaldehyde for at least 24 h. Sections (4-5 *μ*m) on slides were deparaffinized in xylene, rehydrated in decreasing concentrations of ethanol, and stained with hematoxylin and eosin (H&E). All sections were graded blindly by three pathologists under light microscopy according to the following criteria: 0, none; 1, individual cell necrosis; 2, ≤30% lobular necrosis; 3, ≤60% lobular necrosis; 4, >60% lobular necrosis [15].

### 2.6. RNA Isolation and Real-Time PCR Analysis

Total RNA was isolated from the homogenate of the liver with Trizol reagent (Invitrogen) at 12 h after Con A stimulated. Cellular RNA was treated with DNase I and then primed with a dT oligonucleotide and reverse transcribed with Superscript II. For real-time assays, PCR reactions were prepared in SYBR Green PCR Master Mix. DNA targets were amplified and analyzed with a Chromo Real-Time PCR Detection System (Bio-Rad Life Sciences). The murine primer sequences are shown as follows.

Mouse TNF-*α* (Forward, F): 5′-GGGCTACAGG CTTGTCACTCG-3′ and (Reverse, R): 5′-ACTCCAGGCGGTGCCTATGTC-3′), Mouse IL-6 (F): 5′-AGTTGCCTTCTTGGGACTGA-3′ and (R): 5′-TCCACGATTTCCCAGAGAAC-3′, Mouse IFN-*γ* (F): 5′-CCTCAAACTTGGCAATACTCA-3′ and (R): 5′-CTCAA GTGGCATAGATGTGGA-3′), Mouse IL-10 (F): 5′-TGCCACTCAGAA GACTGTGG-3′ and (R): 5′-GTCCTCAGTGTAGCCCAGGA-3′), and Mouse CXCL-10 (F): 5′-TCCAGTTAAGGAGCCCTTTTAGACC-3′ and (R): 5′-TGAAATCATCCCTGCGAGCCTAT-3′ GAPDH (F): 5′-AGAGTGGGAGTTGCTGTTG-3′ and (R): 5′-GCCTTCCGTGTTCCTACC-3′. Total RNA was treated with DNase I to eliminate genomic DNA contamination, followed by synthesis of the first-strand using reverse transcription system. Reverse transcription was carried out as follows: 42°C for 60 min, 70°C for 10 min, and first-strand cDNA was stored at −20°C. Real-time PCR was performed in a 20 *μ*L of reaction solution containing SYBR Premix Ex Taq, primers, and cDNAs. The cycles for PCR were as follows: 95°C for 2 min, 40 cycles of 95°C for 15 s, 58°C for 20 s, and 72°C for 20 s. Melting curves were determined by heat-denaturing PCR products over a 35°C temperature gradient at 0.5°C/s from 65 to 99.5°C. GAPDH was used as an internal control [16]. The relative amount of mRNA was determined using the ΔΔCT technique as described previously [17]. The levels of mRNA were expressed as fold-changes after normalization to GAPDH.

### 2.7. Western-Blotting Analysis of NF-*κ*B

Livers were carefully excised and homogenized into lysis buffer (Thermo, USA) to yield a homogenate. After centrifugation (12000 g for 10 min) at 4°C, protein concentration was detected by Bradford protein assay kit (Thermo, USA) with bovine serum albumin as standard. Equal amounts of protein extracts separated discontinuously onto 10% polyacrylamide gels (Life Technologies, Carlsbad, CA, USA) and transferred to nitrocellulose membranes (Life Technologies). After blockade of nonspecific binding sites, membranes were incubated with various antibodies against I*κ*B*α*, phospho-I*κ*B*α*, NF-*κ*B p65, and phospho-NF-*κ*B p65 (Cell Signaling Technology, Danvers, MA, USA) for 2 h at room temperature. Membranes were developed by chemiluminescence using an Amersham prime ECL Plus detection system (GE Healthcare Life Sciences, Pittsburgh, PA, USA). Signals were densitometrically assessed and normalized to the GAPDH signals [18].

### 2.8. Flow Cytometry Analysis

Single-cell suspensions of blood, liver, and spleen were obtained 12 h after Con A administration. Cells were then stained with fluorescence-labeled antibody (anti-CD4 APC, code: 17-0041-82; clone: GK1.5, eBioscience USA, anti-CD8 PE, code: 11-0081-85; clone: 53-6.7, eBioscience USA). The counts of CD4^+^ and CD8^+^ T lymphocytes infiltrating in the blood, liver, and spleen were analyzed by flow cytometry (Miltenyi, Germany).

### 2.9. Statistical Analysis

All results are expressed as mean ± SD. All statistical analyses were performed by Prism 6.0 (Graph Pad Software, USA). All comparisons among groups were performed by one-way analysis of variance (ANOVA). For multigroup analysis, intergroup comparisons were performed by Dunn's test. *P* < 0.05 was regarded as statistically significant.

## 3. Results 

### 3.1. Salidroside Ameliorates the Injuries of Con A-Induced Hepatitis

**Figure 1:**
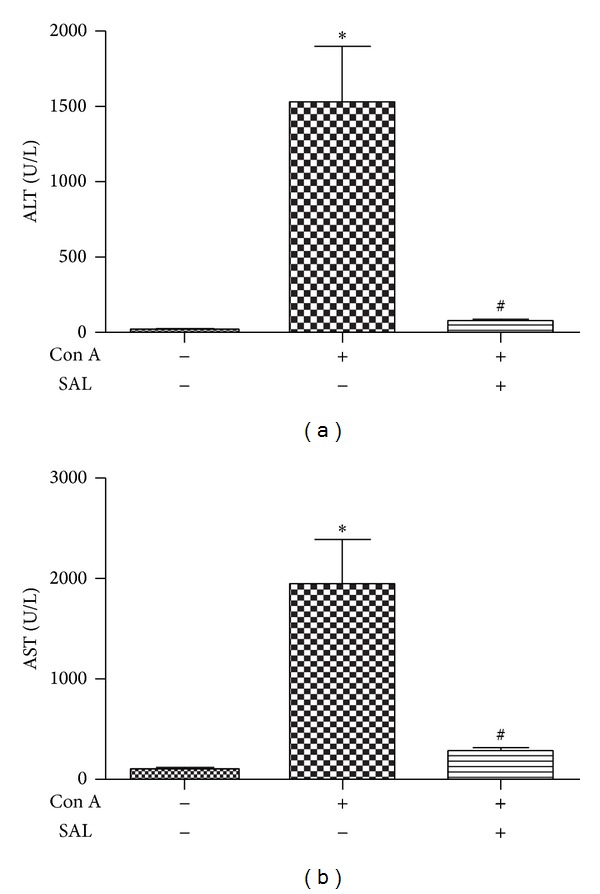
Salidroside pretreatment attenuated liver damage followed Con A administration. (a) and (b) demonstrated salidroside reduced ALT and AST levels in mice 12 h after Con A administration (*n* = 10 mice per group); *P < 0.01 versus control group, ^#^ *P* < 0.01 versus Con A group.

To explore whether salidroside exhibits a protective effect on Con A-induced hepatitis, the experimental hepatitis model on C57BL/6 male mice was established. Compared to control group, plasma ALT and AST levels were elevated excessively in response to Con A administration. A single dose of salidroside pretreatment significantly decreases levels of ALT and AST (*P* < 0.01) (Figures 1(a) and 1(b)), suggesting a protective role of salidroside in Con A-induced hepatitis. We also explored the therapeutic potential of salidroside in Con A-induced hepatitis. Although salidroside decreased levels of ALT and AST slightly, it did not reach a statistical significance (*P* > 0.05, data shown in Figure 8). Accordingly, we determined to explore the effect of pretreatment with SAL in Con A-induced hepatitis.

**Figure 2:**
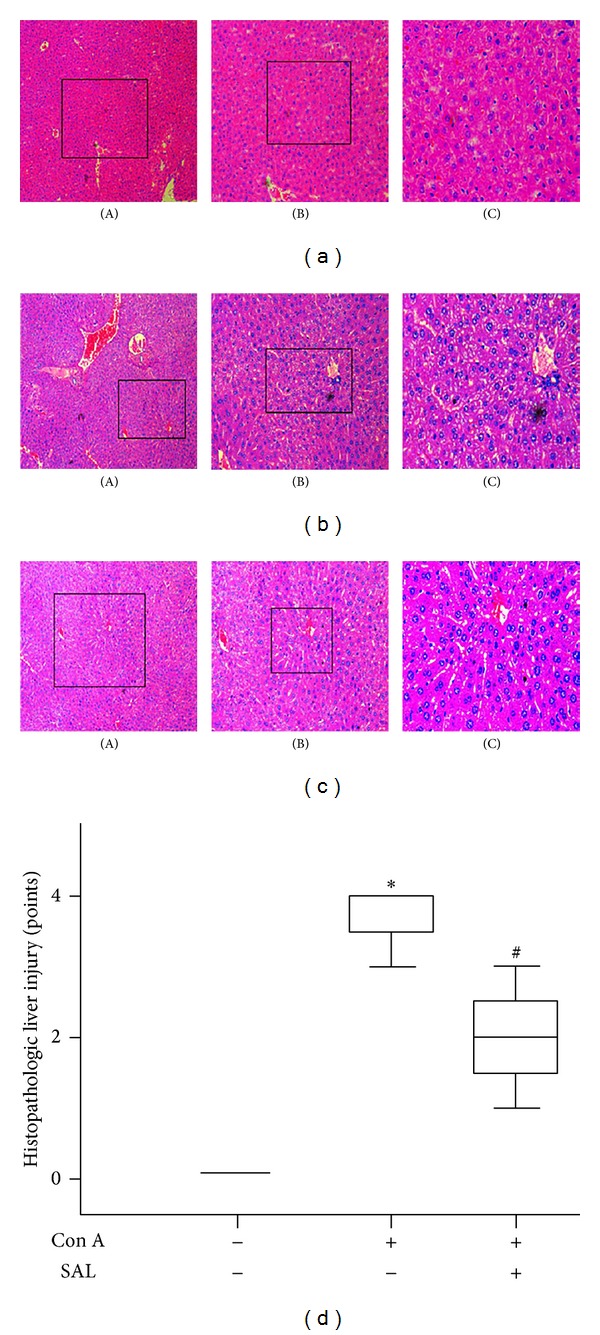
Histopathological changes of mice. The liver tissue sections were stained with H&E. Black squares in (A) were magnified into (B), and blank square in (B) was magnified into (C). Original magnification was ×100 for (A), ×200 for (B), and ×400 for (C). (a) Representative microscopic appearance of liver tissue in control group. (b) Representative microscopic appearance at 12 h after Con A administration. (c) The effect of salidroside on the degrees of liver injury. (d) Severity of all sections was scored blind by three pathologists. *P < 0.01 versus control group, ^#^ *P* < 0.05 versus Con A group. (*n* = 10 mice per group).

The protective effect of salidroside was further confirmed by morphological analysis. After intravenous Con A administration, the liver injury of mice in the Con A group was characterized by massive hepatocyte necrosis and disorder of hepatic sinusoids structure (Figure 2(b)). However, pretreatment with salidroside could alleviate hepatocyte necrosis and T lymphocyte infiltration (Figure 2(c)). The pathological scores showed that liver injury in Con A group was significantly higher than that in salidroside pretreatment group (Figure 2(d)). These results indicated that salidroside is protective against Con A-induced hepatitis.

### 3.2. The Anti-Inflammatory Effect of Salidroside on Con A-Induced Hepatitis

**Figure 3:**
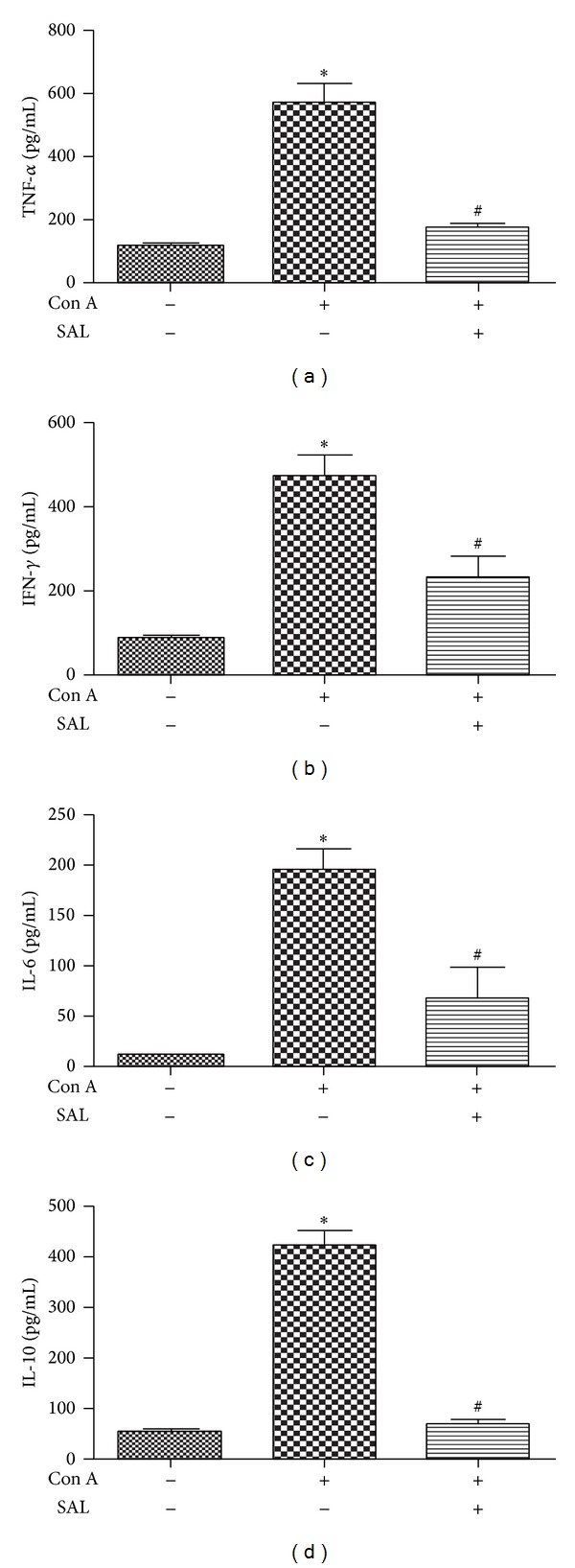
The effect of salidroside on cytokine production in Con A-treated mice. Samples were collected 12 h after Con A administration. (a)–(d) Salidroside reduced plasma proinflammatory cytokine secretion. *P < 0.01 versus control group, ^#^ *P* < 0.01 versus Con A group.

Con A-induced hepatitis is associated with the production of various proinflammatory cytokines. After Con A administration, the levels of proinflammatory cytokines, that is, TNF-*α*, IFN-*γ*, and IL-6, were elevated in the plasma. In the salidroside pretreatment group, the inflammatory cytokines, mentioned above, decreased dramatically at 12 h after Con A administration (*P* < 0.01) (Figures 3(a), 3(b), 3(c), and 3(d)).

**Figure 4:**
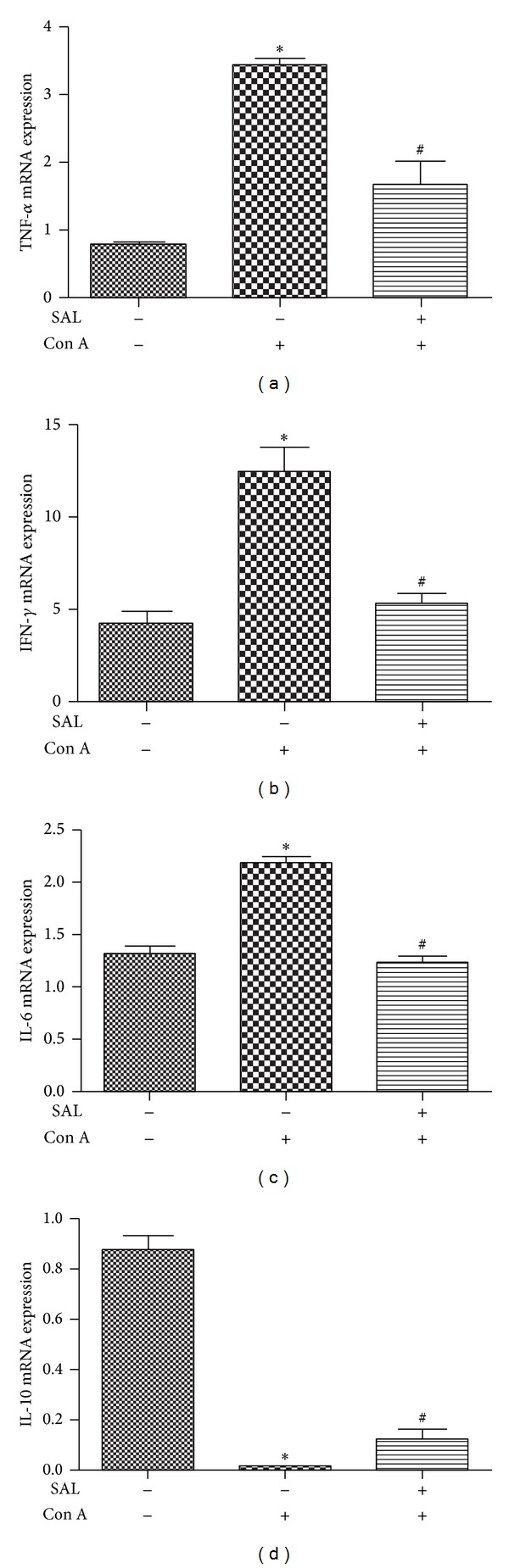
Intrahepatic mRNA expression of cytokines. Samples were collected 12 h after intravenous Con A administration. (a)–(c) Salidroside alleviated intrahepatic mRNA expression of proinflammatory cytokines (TNF-*α*, IFN-*γ*, and IL-6) and (d) Salidroside increased intrahepatic mRNA expression of IL-10. The data output is expressed as a fold-change of expression levels. Level of each type of mRNA from control group was normalized to a value of 1 *P < 0.01 versus control group, ^#^ *P* < 0.01 versus Con A group.

We subsequently investigated the effect of salidroside on expression levels of TNF-*α*, IFN-*γ*, IL-6, and IL-10 mRNA in liver. The results showed that levels of TNF-*α*, IFN-*γ*, and IL-6 mRNA increased in Con A group compared to NS group. Salidroside pretreatment significantly reduced the levels of TNF-*α*, IFN-*γ*, and IL-6 mRNA in the liver (*P* < 0.01) (Figures 4(a), 4(b), and 4(c)). However, IL-10, which is regarded as an anti-inflammation cytokine, and the mRNA level of IL-10 decreased significantly after Con A administration, while salidroside pretreatment can facilitate its secretion (*P* < 0.01) (Figure 4(d)). In addition, the expression level of IL-10 was attenuated by salidroside measured by western blot (Figure 9). Together, these data demonstrated that the protective role of salidroside pretreatment in Con A-induced hepatitis is associated with reduction of TNF-*α*, IFN-*γ*, and IL-6. Oddly, there exist contradictory results of IL-10 in blood measured by ELISA with in liver measured by RT-PCR and western blot. Further studies were needed to clarify the issue.

### 3.3. Salidroside Pretreatment Suppressed Phosphorylation of I*κ*B*α* and p65

**Figure 5:**
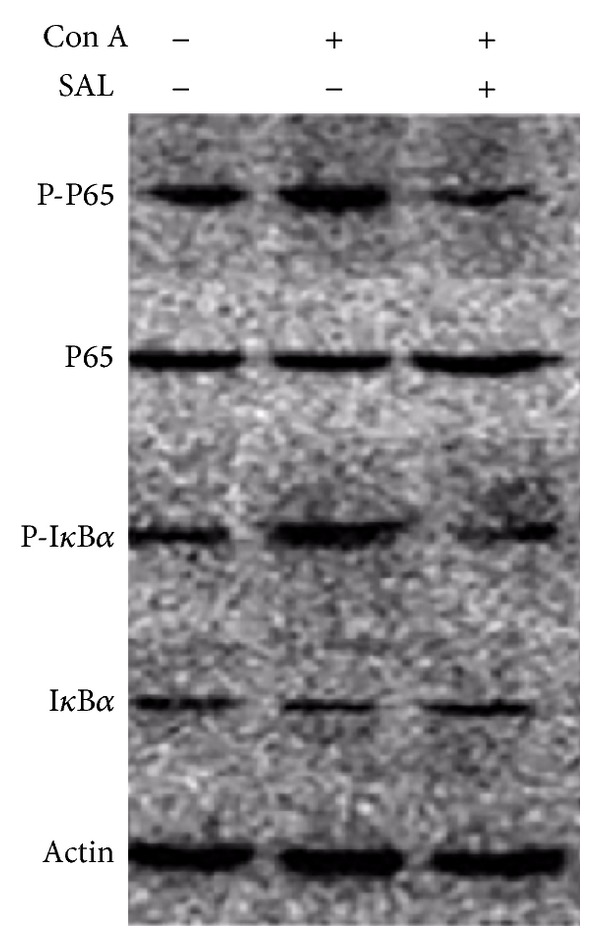
The effect of salidroside pretreatment on NF-*κ*B protein expression. Salidroside pretreatment followed Con A administration attenuated I*κ*B*α* and p65 phosphorylation.

The production of proinflammatory cytokines, including TNF-*α* and IL-6, is largely regulated by the activation of transcription factors such as NF-*κ*B. We then explored whether salidroside inhibits NF-*κ*B activation* in vivo*, which is revealed by the phosphorylation of I*κ*B*α* and p65. Our results found that salidroside pretreatment inhibited phosphorylation of I*κ*B*α* and p65 compared with Con A group (Figure 5).

### 3.4. Salidroside Pretreatment Inhibited CD4^+^ and CD8^+^ T Cells Infiltrating in Liver

**Figure 6:**
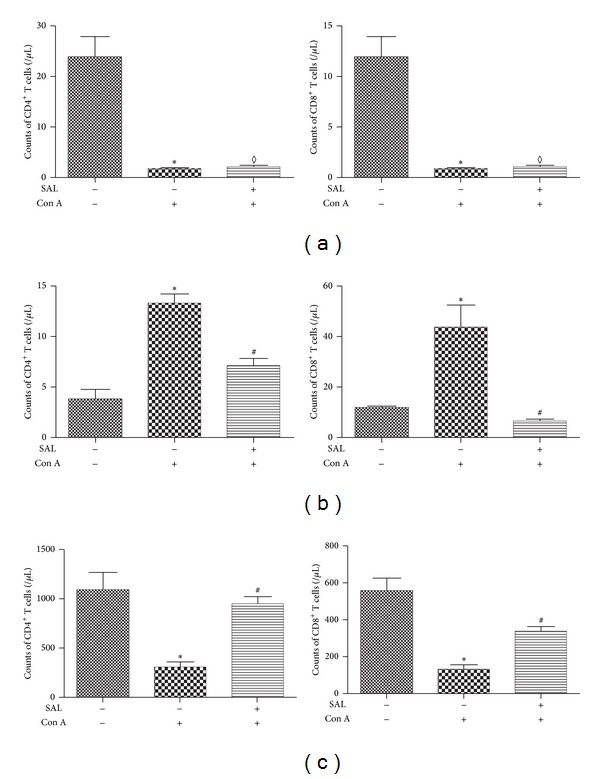
The effect of salidroside on T lymphocytes infiltrated in the blood, liver, and spleen. (a) The number of CD4^+^ and CD8^+^ T lymphocytes in the blood did not show any difference between Con A group and salidroside group. (b) The number of CD4^+^ and CD8^+^T lymphocytes in the liver increased significantly after Con A administration. Salidroside pretreatment reduced the liver-infiltrating CD4^+^ and CD8^+^ T lymphocytes. (c) The number of CD4^+^ and CD8^+^ T lymphocytes in the spleen decreased dramatically after Con A administration; salidroside pretreatment reversed the variation of CD4^+^ and CD8^+^ T lymphocytes recruited to the spleen. *P < 0.01 versus control group, ^#^ *P* < 0.05 versus Con A group, ^*◊*^ *P* > 0.05 versus Con A group.

Previous studies [19, 20] demonstrated that T cells play a critical role in Con A-induced liver injuries. In the present study, we calculated the number and percentage of CD4^+^ and CD8^+^ T lymphocytes in blood, liver, and spleen. Although there was no difference in counts of T lymphocyte in the blood (Figure 6(a)), Con A administration resulted in a prominent increase in the absolute counts of T lymphocyte in the liver. As we expected, salidroside pretreatment decreased the number of T lymphocyte in liver (*P* < 0.01) (Figure 6(b)). We found that Con A administration resulted in a significant decline of T lymphocytes, while salidroside pretreatment increased the counts of T lymphocytes in the spleen (*P* < 0.01) (Figure 6(c)). These data suggest that there might be a shift of T cells from spleen to liver.

### 3.5. Salidroside Pretreatment Inhibited T Cells Infiltration in Liver via CXCL-10

**Figure 7:**
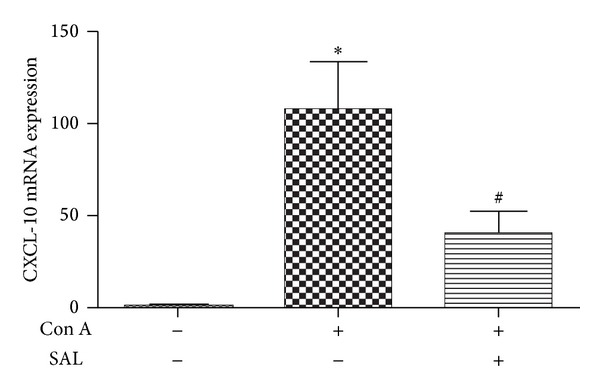
Intrahepatic mRNA expression of CXCL-10. Samples were collected 12 h after Con A administration. Salidroside alleviated intrahepatic mRNA expression of CXCL-10. The data output is expressed as a fold-change of expression levels. Level of each type of mRNA from control group was normalized to a value of 1 *P < 0.01 versus control group, ^#^ *P* < 0.01 versus Con A group.

**Figure 8:**
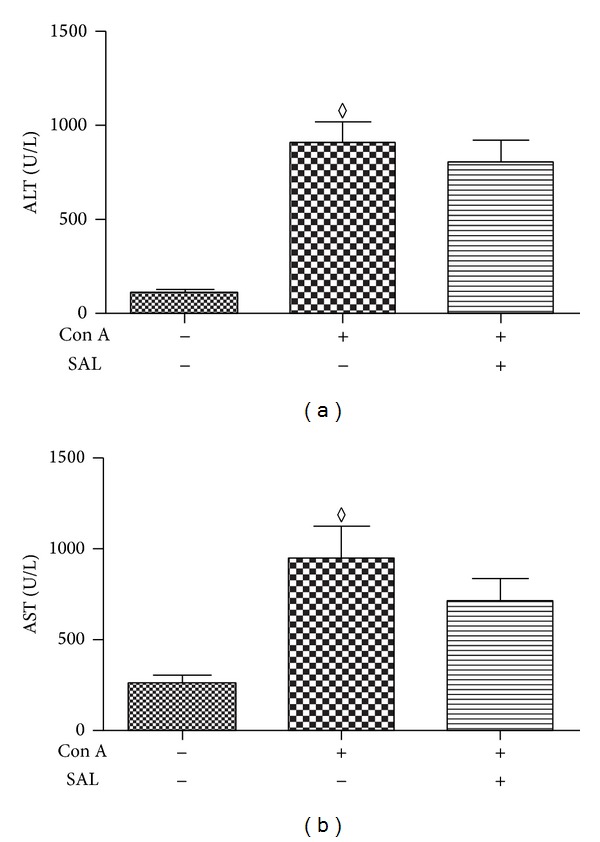
Salidroside cannot attenuate liver damage following Con A administration. (a) and (b) demonstrated that ALT and AST levels did not show any difference between Con A and salidroside groups (*n* = 10 mice per group); ^*◊*^ *P* > 0.05 versus salidroside group.

**Figure 9:**
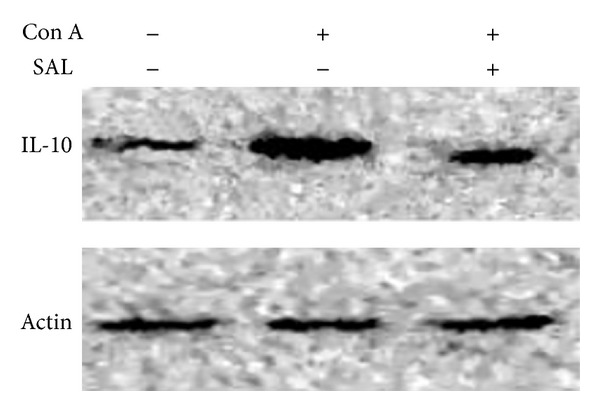
The effect of salidroside pretreatment on IL-10 protein expression. Salidroside pretreatment followed Con A administration attenuated IL-10 protein expression in the liver.

It is well known that hepatic CXCL-10 expression is increased in experimental models of Con A induced liver injury [16] and associated with the migration of T lymphocytes. In the present study, as shown in Figure 7, the expression of CXCL-10 mRNA in the liver was enhanced significantly in Con A group compared with control group (*P* < 0.01), while it was downregulated significantly by salidroside pretreatment (*P* < 0.01). We speculated that the shift of T lymphocytes from spleen to liver was modulated by CXCL-10 partly.

## 4. Discussion 

Hepatitis, caused by virus infection and some certain drugs as well as autoimmunity, is an increasingly frequent health issue. Despite advances in medical science and understanding of pathogenesis of hepatitis, effective therapeutic approaches remained unresolved. In the current study, we investigated the protective effect of salidroside on Con A-induced hepatitis. According to the results of our study, salidroside pretreatment showed a protective effect in Con A-induced hepatitis, reducing the levels of ALT and AST in plasma as well as the severity of hepatic necrosis. The potential mechanisms of its beneficial effects involve inhibiting inflammatory responses and infiltration of lymphocytes. Our study found that salidroside administration after Con A did not show a beneficial effect. Given to the pharmacodynamics and pharmacokinetics of salidroside [21, 22] and Con A [5], salidroside was administrated 1 h before Con A.

Li et al. [23] investigated the effect of salidroside on upregulating the expression of both Th1 and Th2 cytokines, that is, IL-6, TNF-**α*, *and IL-10. Earlier studies demonstrated that TNF-**α** [24] and IFN-**γ** [25] played an essential role in the model of Con A-induced hepatitis. One previous study has shown that TNF-*α* and IFN-*γ* regulated the release of inflammatory cytokines in Con A-induced hepatitis, and IFN^−/−^TNF^−/−^ mice were free from liver injury by Con A administration [26]. In our study, salidroside inhibited the secretion of proinflammatory cytokines, that is, TNF-**α**, IFN-**γ*, *IL-6, and IL-10, as well as their mRNA expression in the liver, except for IL-10. Oddly, the mRNA level of IL-10 in liver was enhanced by pretreatment of salidroside. We speculated that the inflammatory response may be prevented via upregulating the expression of IL-10 partly [27].

It is known that salidroside may be of potential usefulness in the treatment of inflammation. Salidroside exhibits its function partly through modulating the activity of NF-*κ*B [12], which is a nuclear transcription factor regulating the expression of genes involved in inflammation. Our results indicated salidroside pretreatment suppressed I*κ*B*α* kinase and p65 phosphorylation in Con A-induced hepatitis, which in turn inhibited the activation of NF-*κ*B. We speculated that the attenuation of the severity of Con A-induced hepatitis by salidroside pretreatment is mediated by suppression of inflammatory responses through downregulating phosphorylation of NF-*κ*B activity partly.

Immune cells have been documented to be critical for Con A-induced hepatitis, including T lymphocyte, activated natural killer T (NKT) cells, and Kupffer cells [18, 28, 29]. Among these effector cells, CD4^+^ T lymphocytes and cytotoxic CD8^+^ T lymphocytes (CTL) are the predominant cells infiltrated into the liver [12]. In our study, we found that the number of CD4^+^ and CD8^+^ T lymphocytes located in the liver was increased dramatically after Con A injection. Salidroside pretreatment was associated with a remarkable decrease of CD4^+^ and CD8^+^ T lymphocytes. In contrast, the total number of CD4^+^ and CD8^+^ T lymphocytes counted in the spleen differed from that in the liver. Lymphocytes in liver can be classified into two groups, exogenous and endogenous [30]. Exogenous lymphocytes partially originate from the spleen [31] and enter the liver through circulation. However, the difference of lymphocytes in the blood of the present study was not significant among groups. We speculated that it might be just in balance of the lymphocytes at the checking time. Further studies were needed to clarify the issue.

**Figure 10:**
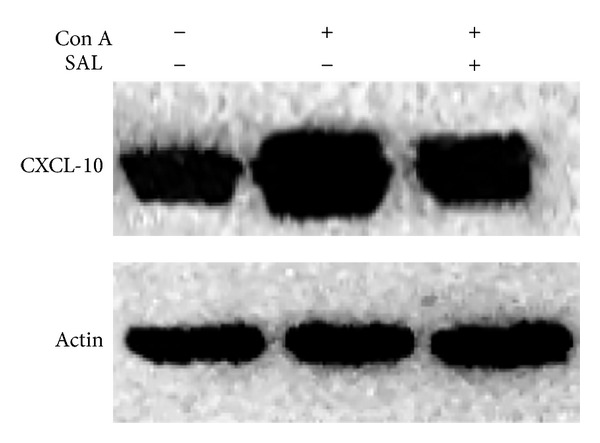
The effect of salidroside pretreatment on CXCL-10 protein expression. Salidroside pretreatment followed Con A administration attenuated CXCL-10 protein expression in the liver.

One previous study demonstrated that CXCL-10 expression was increased in experimental models of Con A induced liver injury [16]. CD4^+^ T cells infiltration in the liver had been confirmed caused by CXCL-10 [16, 18, 32–34]. Recruited T lymphocytes may be responsible for enhanced IFN-*γ* and TNF-*α* production, which in turn stimulates CXCL-10 secretion from a variety of cells, creating an amplification feedback loop [35]. As demonstrated in our study, salidroside downregulated the CXCL-10 mRNA expression in the liver induced by Con A. In addition, the protein level of CXCL-10 also changed as same as its mRNA change (Figure 10). Taken together, CXCL-10 expression may contribute to accumulation of T lymphocytes and subsequent liver injury in Con A-induced hepatitis.

These results indicate that salidroside may become a new agent for liver hepatitis prevention. However, it should be noted firstly that our study is limited by the lack of investigation of other pharmacological actions of salidroside, such as its antioxidant and other properties, which may also be involved in Con A-induced liver injury. Secondly, only one dose of salidroside was used in the study, which may ignore its dose dependent effect. Thirdly, samples were harvested all at one time point, which may limit the protective effect of salidroside. Fourthly, NF-*κ*B is a general signaling pathway in modulating the effect of salidroside on Con A model, which may not explain the mechanism clearly. Finally, T lymphocyte infiltration and inflammation response were attenuated by salidroside. However, the effect of salidroside on proliferation and activity of T lymphocyte especially on regulatory T cells needs further studies.

## 5. Conclusions

Taken together, our study indicated that salidroside pretreatment ameliorates Con A-induced hepatitis and suggested that infiltrated T lymphocytes modulated by CXCL-10 and NF-*κ*B signaling pathway served to attenuate the inflammatory response. These findings raised the promising potential of developing novel pharmacological treatments for T cell mediated liver diseases.
